# Comparative Protective Effects of Static Magnetic Field-Treated and Untreated Corn Sprouts on DSS-Induced Ulcerative Colitis in Mice: Inflammation Modulation and Gut Microbiota Regulation

**DOI:** 10.3390/foods14183248

**Published:** 2025-09-18

**Authors:** Jiaqi Zhao, Ye Gu, Shijie Sun, Aoran Guo, Mingzhu Zheng, Dan Cai, Ke Lin, Huimin Liu

**Affiliations:** 1 College of Food Science and Engineering, Jilin Agricultural University, Changchun 130118, China; 15774902507@163.com (J.Z.); 19969512361@163.com (Y.G.); sunshijie77@163.com (S.S.); 15633966010@163.com (A.G.); zhengmingzhu@jlau.edu.cn (M.Z.); caidan@jlau.edu.cn (D.C.); 2 National Engineering Research Center of Wheat and Corn Deep Processing, Changchun 130118, China

**Keywords:** static magnetic field, corn sprouts, ulcerative colitis, anti-inflammation, gut microbiota

## Abstract

Static magnetic field (SMF) is an emerging food-processing technology that has been widely applied in areas such as processing and sterilization. However, its influence on sprout production or health-related attributes has not yet been investigated. Therefore, in this study, corn sprouts were used as the raw material to compare the differential health effects of SMF treatment in a mouse model of dextran sulfate sodium (DSS)-induced colitis. The in vivo anti-inflammatory effects of SMF-treated corn sprouts were assessed by analyzing changes in their active ingredients. Histological staining, qRT-PCR and 16s rDNA sequencing were performed in the DSS-induced colitis mouse model. The results indicated that dietary fiber and total phenol contents were significantly higher in SMF-treated corn sprouts (M-CSP) compared to SMF-untreated corn sprouts (C-CSP). M-CSP alleviated the symptoms of DSS-induced colitis, significantly reduced colonic epithelial damage, and suppressed the secretion of pro-inflammatory factors. In addition, M-CSP markedly improved the diversity and abundance of intestinal microbiota. These findings provide new insights for the development and application of SMF technology to functional food ingredients.

## 1. Introduction

Sprouts, plant-based foods rich in nutrients and bioactive components, have received widespread attention in the food and health fields in recent years. Among these, corn sprouts, a nutrient-rich vegetable resource, have attracted considerable attention because of their high contents of carotenoids, total phenols, free phenols, and binding phenols [1]. Research indicates that corn sprouts contain higher levels of carotenoid content than raw seeds [2]. In addition, moderate consumption of corn sprouts may slow aging, prevent heart disease, and combat eye aging. Currently, a major challenge faced in the sprout industry is improving seed germination ability for rapid growth of sprouts while improving nutrient content. To address this, corn seeds have been treated using physical processing methods such as plasma [3], microwave [4], ultrasound [5] and static magnetic field (SMF) [6]. These emerging methods are environmentally efficient and sustainable.

SMF treatment has been widely used as a physical method to improve plant germination, growth, and development. Recent studies have reported that appropriate SMF treatment increases germination rates in flaxseeds [7], soybeans [8], and brown rice [9]. Piacentini et al. demonstrated that SMF delayed senescence and increased the levels of antioxidant enzymes in yellowing cucumber seedlings [10], while Sujak et al. [11] observed increased crude fiber content in amaranth after SMF treatment. SMF treatment was found to affect active fractions of corn sprouts and significantly increase dietary fiber content. Studies have demonstrated that SMF treatment renders the structure of dietary fibers loose and porous [12]. Consequently, these structural modifications promoted the release of polyphenolic compounds encapsulated within the fiber matrix. Therefore, we hypothesized that SMF-treated corn sprouts would exhibit enhanced bioactivity.

Ulcerative Colitis (UC), a chronic inflammatory bowel disease (IBD) alongside Crohn’s disease (CD) [13], is characterized by subtle and nonspecific symptoms primarily affecting the colon [14]. The main clinical features include abdominal pain, diarrhea, stool blood, inflammatory infiltration of the intestinal epithelium, and intestinal mucosal damage [15]. Biological treatment therapies for ulcerative colitis (UC) target specific immune pathways, such as Anti-TNFα agents Infliximab and Adalimumab, Anti-integrin agent Vedolizumab, and IL-12 inhibitor Ustekinumab [16]. However, these treatments have limitations such as variable patient responses to drug treatment, high toxicity, side effects, and high costs [17]. Hence, researchers have focused on natural bioactive compounds and functional foods, such as dietary fiber [18], polyphenols [19] and carotenoids [20].

Our previous study reported that SMF treatment increases endogenous enzyme activity in corn sprouts and enhances the antioxidant activity of dietary fiber [12]. Building on these findings, this study explored the health effects of SMF-treated corn sprouts (M-CSPs) to support the development of new sprout-based health foods. Moreover, corn sprouts were used as the research material. This study aimed to examine the effects of SMF treatment on the active components of corn sprouts and to verify their health efficacy in a mouse model of colitis. Histological staining, qRT-PCR, and 16s rDNA sequencing analyses were conducted to elucidate the mechanisms of M-CSP in UC treatment.

## 2. Materials and Methods

### 2.1. Preparation of M-CSP and Static Magnetic Field-Untreated Corn Sprouts (C-CSP)

M-CSP was prepared according to a previously reported methodology [12]. Briefly, corn seeds were treated with a magnetic induction intensity of 30 mT for 2 h (MF1-T2, high- and low-temperature SMF test chambers, Wuxi, China) and incubated in a plant growth chamber at a temperature of 25 °C and humidity of 70–80% for 7 days to obtain corn sprouts (taking the above-ground parts). C-CSP was prepared under identical handling without magnetic exposure. The lyophilized corn sprouts were crushed, sieved through a 200-mesh sieve, and stored in a desiccator protected from light.

### 2.2. Determination of the Bioactive Components in Corn Sprouts

The nutritional components of corn sprouts, including protein, fat, moisture, ash, dietary fiber, soluble sugar and starch were analyzed in accordance with the following Chinese national standards: GB 5009.5-2016, GB 5009.6-2016, GB 5009.3-2016, GB 5009.4-2016, GB 5009.88-2014, GB/T 37493-2019 and GB 5009.9-2023 [21,22,23,24,25,26,27]. Total phenolic content was determined according to the method with minor modifications [28]. Briefly, mix 200 μL of free or bound phenolic extract with 1.5 mL of Folin–Ciocalteu working solution (diluted 10-fold, incubate at room temperature protected from light for 5 min), then add 1.5 mL of 7.5% Na_2_CO_3_ solution and incubate at 30 °C protected from light for 1 h. Measure the absorbance at 765 nm. Total phenolic content was calculated from the standard curve using gallic acid as the standard.

### 2.3. Construction and Treatment of DSS

Twenty-four male C57BL/6N mice (18 ± 2 g body weight) were obtained from Viton Lever Laboratory Animals Ltd. (Beijing, China). Mice were maintained in a pathogen-free environment on a 12/12-h light/dark cycle. All experiments complied with national guidelines “Regulations for the Administration of Affairs Concerning Experimental Animals.” The experiments were approved by the Use Committee of the Jilin Academy of Chinese Medicine Sciences (JLSZKYDWLL2023-057).

After seven days of acclimatization, the mice were randomly assigned to four groups: Con, DSS, C-CSP, and M-CSP (*n* = 6). The Con and DSS groups were fed a normal AIN93G diet, while the other groups received customized feed with 5% C-CSP or M-CSP added to 1 kg of AIN93G feed. The feed formulae are listed in Table S1. After acclimatization, the drinking water for the DSS, C-CSP, and M-CSP groups was changed to a 3% DSS solution configured with sterile water to establish the UC model. The Con group was maintained in sterile water until use. The other groups received equivalent amounts of distilled water daily at pre-determined intervals. Body weights of mice were monitored daily. Finally, the mice were fasted for 12 h and euthanized via cervical dislocation. Fecal occult blood tests were performed before dissection. Colons were dissected and isolated, and major organs (heart, liver, spleen, lung, and kidney) were weighed and recorded. Blood and stool samples were collected. The mouse colons were divided into two sections: one was fixed in 4% paraformaldehyde solution for tissue sectioning, histopathological observation, and scoring, and the other was placed in 1.5 mL enzyme-free EP tubes, flash-frozen in liquid nitrogen, and stored at −80 °C for molecular index detection.

### 2.4. Evaluation of Disease Activity Index (DAI)

Daily weight loss, fecal morphology, and occult blood were recorded. The DAI was calculated according to the evaluation criteria and formulas, with the scoring criteria shown in Table S2. DAI assessments were independently conducted by two blinded pathologists to the treatment identities, and discrepancies were resolved by a consensus review.

(1) $$\begin{array}{c} DAIScore=Body\;Weight\;Loss\;Score+fecal\;morphology\;Score\\ +Fecal\;Blood\;Score\;\;\;\;\;\; \end{array}$$

### 2.5. Evaluation of Organ Index

The organs were weighed to calculate the corresponding organ indices. The following formula was used to compute the organ index.

(2) Organ index(mg/g)=(Organ weight×1000)/weight(g)

### 2.6. Histopathological Analysis

Colon tissues were extracted from physiological vials containing paraformaldehyde. The mixture was washed with flowing water for 5 h. Cut tissues were subjected to sectioning, xylene washing, ethanol gradient dehydration, and wax dipping. Tissue sections were processed for hematoxylin and eosin staining. Histological changes in the H&E-stained sections were assessed and imaged using the NIS-ELEMENTS BR Imaging Analysis System (Tokyo, Japan). Histological damage to the colonic tissue was assessed based on inflammation, mucosal damage, degree of glandular damage, and the extent of the affected area (Table S3) [29]. Histopathological scoring was performed by independent experts under double-blind conditions.

### 2.7. Determination of Pro- and Anti-Inflammatory Cytokines in Colon Tissues

ELISA kits (Shanghai Enzyme Linked Biotechnology Co., Ltd., Shanghai, China) were used to measure the amounts of TNF-α, IL-6, IL-10, and IL-1β. Blood samples were stored in a refrigerator at 4 °C overnight, coagulated, and centrifuged at 4000 rpm for 10 min at 4 °C (HC-3618R High-Speed Refrigerated Centrifuge, Hefei, China), followed by ELISA analysis using the top layer of serum.

### 2.8. Determination of Nitric Oxide (NO) and Malondialdehyde (MDA) in Colon Tissues

To prepare colon tissue homogenate, murine colon tissue was collected and weighed, then combined with PBS at a 1:9 *w/v* ratio, and homogenized using a frozen tissue grinder (TissuePrep™ TP-24 Tissue and Cellular Homogenizer, Shanghai, China). The sample was centrifuged at 12,000 rpm for 15 min at 4 °C (HC-3618R High-Speed Refrigerated Centrifuge, Hefei, China), followed by subsequent harvesting of the supernatant. NO and MDA levels in the supernatant were measured using commercial detection kits (Nanjing Jiancheng Bioengineering Institute Co., Ltd., Nanjing, China).

(3) $$NO\left(\frac{\mu mol}{gprot}\right)=\frac{A_{2}-A_{0}}{A_{1}-A_{0}}\times C_{s}\times N÷C_{pr}$$

*A*_0_, blank absorbance; *A*_1_: Standard absorbance; *A*_2_: Test sample absorbance; *Cs*: Standard concentration; *N*, dilution multiples of colonic tissue supernatant prior to the assay; *C_pr_*: Protein concentration (mg prot/mL) in the homogenate at equivalent dilution.

(4) $$MDA\left(\frac{nmol}{mgprot}\right)=\frac{A_{2}-A_{0}}{A_{1}-A_{0}}\times C_{S}\times N$$

*A*_0_, blank absorbance; *A*_1_: Standard absorbance; *A*_2_: Test sample absorbance; *Cs*: Standard concentration; and *N*, dilution multiples of serum samples.

### 2.9. Determination of Serum Superoxide Dismutase (SOD)

Serum samples were diluted to 1:15 in PBS prior to analysis. Serum SOD levels were measured using a commercial detection kit (Nanjing Jiancheng Bioengineering Institute Co., Ltd., Nanjing, China).

(5) $$SOD\left(\frac{U}{mL}\right)=\frac{A_{0}-A_{1}}{A_{0}}÷50\%\times N_{C}\times N$$

*A*_0_, reference absorbance (control); *A*_1_, absorbance of the test sample; *Nc*, dilution multiples of the reaction system; *N*, predilution factor of the serum sample.

### 2.10. qPCR Analysis of NF-κB Pathway Genes

Total RNA was extracted from the colon tissue using Trizol reagent (Takara, Dalian, China). Next, the Prime Script RT reagent kit (Takara, Japan) was used to synthesize complementary cDNA, and the obtained cDNA was stored at −20 °C. After setting up the real-time fluorescent reaction mixture, the cDNA was amplified by PCR (Eppendorf^®^ PCR Thermal Cycler, Hamburg, Germany). Finally, the amplified products were analyzed (Agilent Mx3000P Real-Time PCR System, Agilent Technologies, Santa Clara, CA, USA). Table S4 lists the precise primers utilized. The thermal cycling protocol comprised initial denaturation at 95 °C for 30 s, 40 cycles of denaturation at 95 °C for 10 s, annealing at 60 °C for 30 s, extension at 72 °C for 30 s, and melting curve analysis following amplification at 95 °C for 1 min, 55 °C for 30 s, and 95 °C 30 s.

### 2.11. High-Throughput Sequencing of Fecal 16s rDNA in Mice

Mouse fecal colony DNA was extracted using the QIAamp Fast DNA Stool Mini Kit (Hilden, Germany). After DNA extraction, the concentration and purity of the DNA were evaluated using a NanoDrop apparatus (Illumina NovaSeq 6000 sequencing system, Illumina, San Diego, CA, USA). PCR was performed to amplify the V3-V4 region of the 16S rDNA gene (Bio-Rad T100 Gradient Thermal Cycler, Hercules, CA, USA). Sequencing was conducted by Tianjin Novogene Co., Ltd. (Tianjin, China) using the Novaseq-PE250 platform.

### 2.12. Statistical Analysis

All data are presented as mean ± standard deviation (SD). Statistical analyses were performed using GraphPad Prism 9.0.0 GraphPad software. Statistical significance among groups was compared via either Student’s *t*-test or ANOVA, followed by Duncan’s multiple range test. *p* < 0.05 was considered statistically significant.

## 3. Results

### 3.1. Effects of SMF Treatment on Bioactive Components in Corn Sprouts

The effects of SMF treatment on the bioactive components of corn sprouts are shown in Table 1. Compared to the C-CSP, SMF treatment resulted in a slight reduction in protein content and a significant decrease in fat content, attributable to lipid catabolism, which provides energy and biosynthetic precursors during sprout growth. Concurrently, compared with the C-CSP group, M-CSP exhibited elevated soluble dietary fiber (7.33%) and insoluble dietary fiber (1.90%), with free phenols at 1.86 mg/g (24.00% increase) and bound phenolics at 0.48 mg/g (81.68% increase).

### 3.2. Effect of M-CSP on Clinical Symptoms in DSS Mice

**Figure 1:**
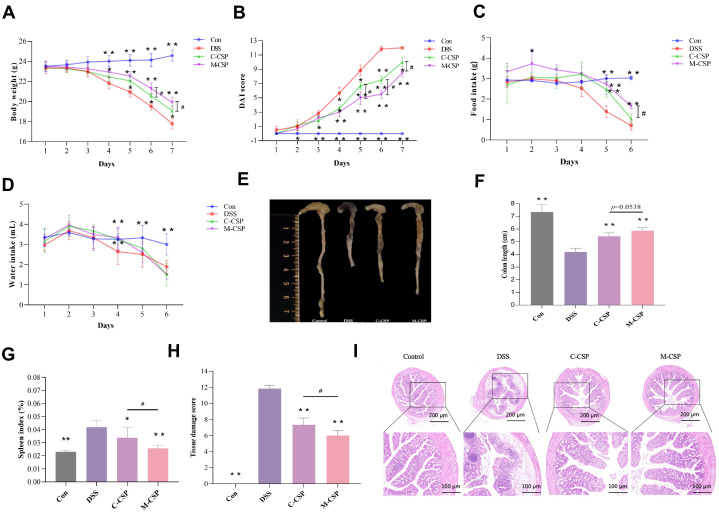
M-CSP supplementation improved the symptoms of DSS-induced colitis. (**A**) Body weight of mice. (**B**) DAI score. (**C**) Food intake and (**D**) water intake of mice. (**E**) Colon appearance of mice. (**F**) Colon length. (**G**) Spleen index. (**H**) Colonic tissue injury score. (**I**) H&E staining. Data represent mean ± standard deviation (*n* = 6). * *p* < 0.05 and ** *p* < 0.01 vs. DSS groups. Significant difference between C-CSP and M-CSP groups (^#^ *p* < 0.05).

The ameliorative effect of M-CSP on colitis was evaluated in a mouse model of DSS-induced UC. As shown in Figure 1A, compared to the Con group, the DSS group started to lose weight significantly from day 4 onwards and developed diarrhea. On day 7, they exhibited blood-watery feces, severe redness, and perianal swelling, demonstrating that UC was successfully induced by DSS. Compared with the DSS group, the M-CSP group showed a significant increase in weight (*p* < 0.01). Moreover, the DAI scores decreased significantly after M-CSP treatment (*p* < 0.01) (Figure 1B). In this study, colitis was modeled by ingesting sterilized water with 3% DSS; therefore, the extent of colitis modeling in mice was directly related to the amount of water ingested. In contrast to the DSS group, the decreasing trend of water and food intake of mice in the C-CSP and M-CSP groups was slower (Figure 1C,D), indicating that CSP alleviated colitis-associated appetite loss, with M-CSP was more effective than C-CSP.

As shown in Figure 1E,F, colon length in the DSS group (4.18 cm) was significantly shorter than that in the Con group (7.35 cm) (*p* < 0.01), indicating successful establishment of the UC model. Compared with the DSS group, colon lengths in the C-CSP (5.43 cm) and M-CSP groups (5.87 cm) were significantly increased (*p* < 0.01), and M-CSP significantly alleviated the shortened colon lengths of UC mice compared with C-CSP. Collectively, these results demonstrate that M-CSP effectively ameliorated the symptoms of colitis.

### 3.3. Effect of M-CSP on Histologic Changes in DSS Mice

Changes in immune organ indices indirectly reflect the degree of inflammatory reactions. As shown in Table 2, compared to the Con group, spleen indices of the DSS group were significantly increased (*p* < 0.01), indicating that DSS can cause injury to the spleen. These findings are consistent with previously reported results [30]. Compared with the DSS group, intervention with M-CSP significantly reduced the DSS-induced increase in spleen index (*p* < 0.01). These results indicate that M-CSP has a positive effect on alleviating immune dysregulation caused by UC.

Colitis is associated with significant mucosal damage, decreased goblet cell counts, and inflammation. The colonic mucosa of mice in the Con group was intact, with a flat surface, no villi, an orderly arrangement of glandular cells, moderate crypt depth, and no inflammatory edema or lesions. After DSS induction, the colonic mucosa of the mice showed irregularity; the intestinal wall was thinned; the lesions infiltrated the muscularis propria; most areas exhibited loss of glands, crypt structure, and cup cells; and the intestinal tissues showed obvious swelling (Figure 1I), indicating successful establishment of the colitis model. In the C-CSP and M-CSP groups, some areas of the mucosal layer showed epithelial cell detachment, localized thinning of the intestinal wall, connective tissue hyperplasia in the lamina propria, signs of infiltration by diseased cells, and apparent localized lesions. In the M-CSP group, some areas of intestinal gland spacing were mildly hyperplastic with a more normal structural morphology, and no obvious lesions or inflammatory changes were observed. Tissue damage scores were significantly increased (Figure 1H, *p* < 0.01), indicating that both C-CSP and M-CSP ameliorated colonic tissue damage in UC mice, with M-CSP being more effective in alleviating DSS-induced colonic tissue damage than C-CSP.

### 3.4. Effects of M-CSP on Oxidative Stress-Related Indices and Inflammatory Factors in DSS Mice

**Figure 2:**
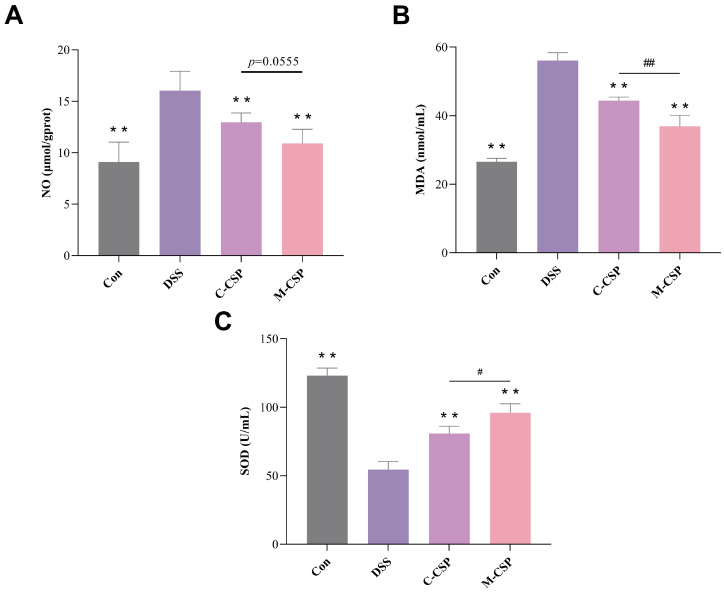
Effect of M-CSP on redox level in colitis mice. (**A**) NO content in the colon tissue of mice. (**B**) MDA content in the serum of mice. (**C**) SOD activity in serum of mice. Data represent mean ± standard deviation (*n* = 6). ** *p* < 0.01 vs. DSS groups. Significant difference between C-CSP and M-CSP groups (^#^ *p* < 0.05 and ^##^ *p* < 0.01 ).

Excessive oxidative stress is associated with cellular damage [31]. As shown in Figure 2, compared to the DSS group, NO levels in the C-CSP and M-CSP groups significantly decreased by 19.15% and 32.00% (*p* < 0.01), respectively (Table S5). Serum MDA levels in the C-CSP and M-CSP groups significantly decreased by 20.89% and 34.23%, respectively (*p* < 0.01) (Table S5). SOD levels increased significantly by 48.58% and 76.16%, respectively (*p* < 0.01) (Table S5), indicating that C-CSP and M-CSP alleviated oxidative stress in mice with colitis. The antioxidant capacity of M-CSP was higher than that of C-CSP (*p* < 0.05). Thus, M-CSP was more effective in relieving oxidative stress in UC mice.

**Figure 3:**
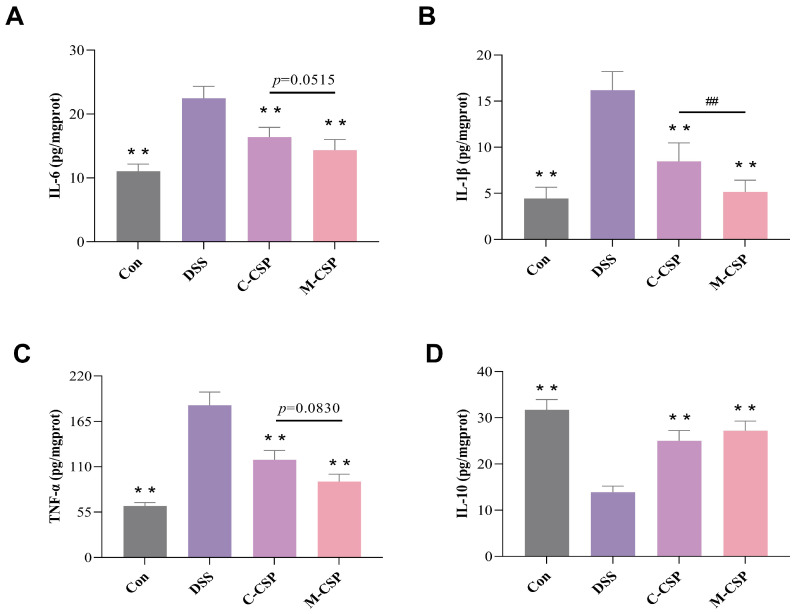
M-CSP treatment modulated cytokine expression in UC mice. (**A**) IL-6. (**B**) IL-1β. (**C**) TNF-α. (**D**) IL-10. Data represent mean ± standard deviation (*n* = 6). ** *p* < 0.01 vs. DSS groups. Significant difference between C-CSP and M-CSP groups (^##^ *p* < 0.01).

IL-10 is an anti-inflammatory cytokine whose production reduces by worsening inflammation. IL-6, IL-1β and TNF-α are pro-inflammatory cytokines highly produced in UC, exacerbating inflammation. As depicted in Figure 3, compared with the DSS group, the levels of IL-6, IL-1β and TNF-α were significantly decreased (*p* < 0.01) after C-CSP and M-CSP interventions by 27.09% and 36.21%, 47.65% and 68.21%, and 50.18% and 57.33%, respectively (Table S6). Meanwhile, IL-10 levels significantly increased (*p* < 0.01) by 80.07% and 95.60%, respectively (Table S6). In conclusion, C-CSP and M-CSP demonstrated strong anti-inflammatory effects, with M-CSP exhibiting more pronounced activity.

### 3.5. Effect of M-CSP on Myeloperoxidase (MPO), COX-2 and iNOS mRNA Expression

**Figure 4:**
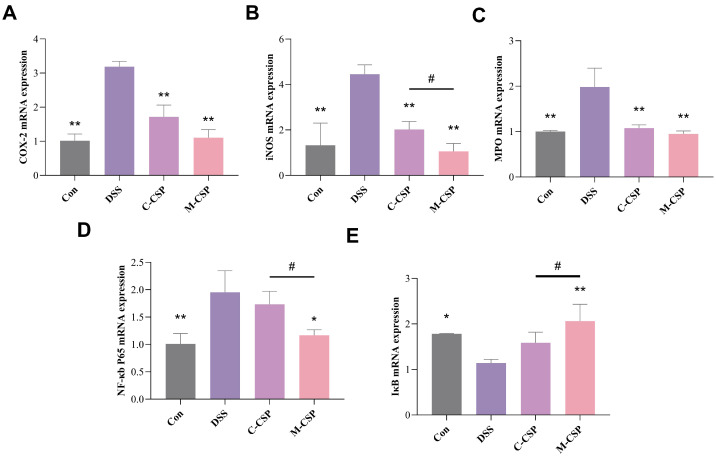
Expression analysis of NF-κB pathway-related genes in the colon. The mRNA expression levels of COX-2 (**A**), iNOS (**B**), MPO (**C**), NF-κb p65 (**D**) and IκB (**E**) in the colorectal region as determined by qRT-PCR. Data represent mean ± standard deviation (*n* = 6). * *p* < 0.05 and ** *p* < 0.01 vs. DSS groups. Significant difference between C-CSP and M-CSP groups (^#^ *p* < 0.05).

Activated NF-κb promotes transcriptional upregulation of several genes involved in almost every aspect of immune response, including iNOS and COX-2 [32]. MPO activity is a crucial marker of neutrophil infiltration and inflammation. Consequently, qPCR was used to detect mRNA expression levels of COX-2, iNOS, and MPO. As shown in Figure 4A–C, COX-2, iNOS, and MPO mRNA levels were significantly higher in the DSS group (*p* < 0.01). However, these changes were significantly suppressed in the C-CSP and M-CSP groups (*p* < 0.05). M-CSP showed a more significant inhibitory effect on inhibiting production (*p* < 0.05), indicating effective anti-inflammatory activity and reduced pro-inflammatory responses induced by DSS.

### 3.6. Effect of M-CSP on the NF-κb Signaling Pathway

The NF-κb pathway plays a vital role in the pathogenesis and progression of IBD [33]. NF-κb raises inflammatory factor levels via conventional and non-traditional routes [34], and its activity is regulated by NF-κb inhibitors IκB. NF-κb p65 expression was detected using qPCR. As shown in Figure 4D,E, NF-κb p65 mRNA expression in colon tissue was significantly increased in the DSS group (*p* < 0.01), suggesting an inflammatory state and activation of inflammation-associated signaling pathways. In the M-CSP group, mRNA expression of NF-κb p65 decreased significantly (*p* < 0.01), while IκB levels increased significantly (*p* < 0.05). These results suggest that M-CSP attenuates NF-κb p65 activation and the subsequent inflammatory cascade.

### 3.7. Effect of M-CSP on Intestinal Barrier Integrity in DSS Mice

**Figure 5:**
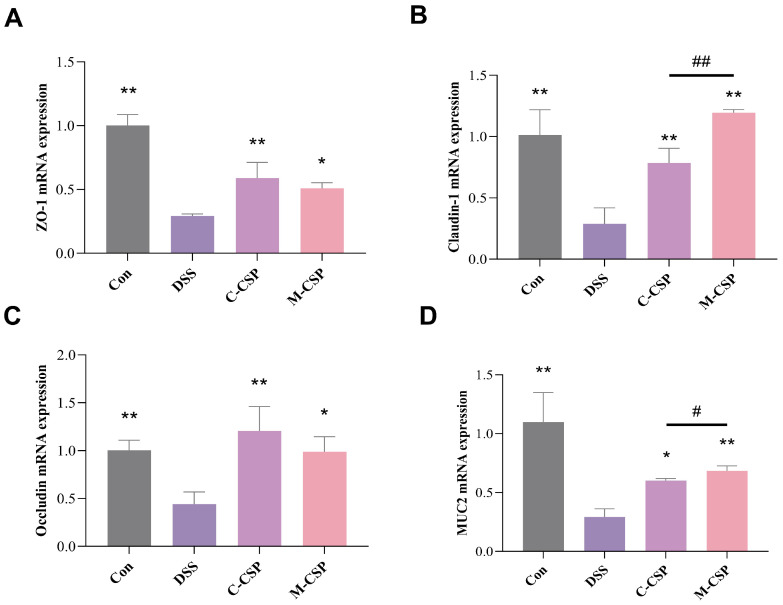
Effect of M-CSP on intestinal barrier integrity in DSS mice. The mRNA expression levels of ZO-1 (**A**), Claudin-1 (**B**), Occludin (**C**) and MUC2 (**D**) in colorectal region. Data represent mean ± standard deviation (*n* = 6). * *p* < 0.05 and ** *p* < 0.01 vs. DSS groups. Significant difference between C-CSP and M-CSP groups (^#^ *p* < 0.05 and ^##^ *p* < 0.01).

Decrease in tight junction proteins increases the risk of colitis [35]. As shown in Figure 5, mRNA expression levels of ZO-1, Claudin-1, Occludin and MUC2 significantly decreased in the DSS group (*p* < 0.01), suggesting that DSS treatment disrupted the permeability of the intestinal barrier. However, M-CSP supplementation increased the expression of these mRNA (*p* < 0.05). These results suggested that M-CSP effectively restored DSS-induced changes in colonic permeability.

### 3.8. Effects of M-CSP on Gut Microbiota Composition in DSS Mice

**Figure 6:**
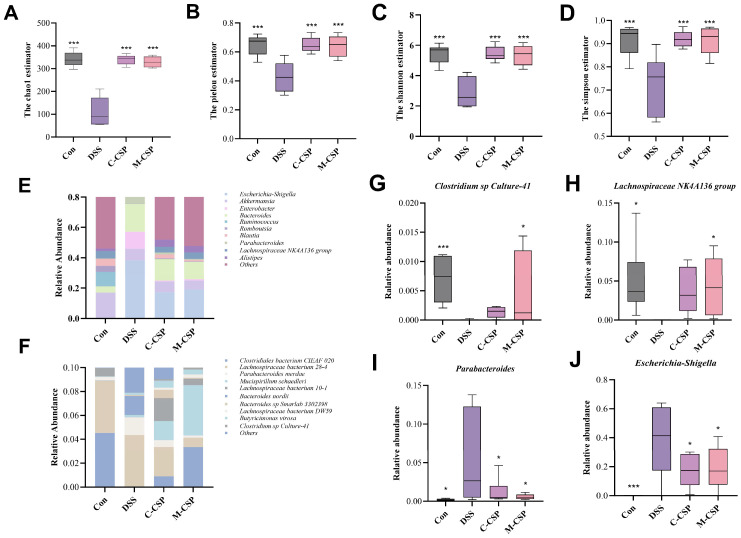
Effects of M-CSP on gut microbiota composition in UC mice. α-diversity indices: (**A**) Chao1, (**B**) Pielou, (**C**) Shannon and (**D**) Simpson. (**E**) Top 10 genera by relative abundance. (**F**) Top 10 species by relative abundance. Quantitative relative abundance: (**G**) *Clostridium sp Culture-41*, (**H**) *Lachnospiraceae NK4A136 group*, (**I**) *Enterobacter*, (**J**) *Escherichia-Shigella*. Data represent mean ± standard deviation (*n* = 6). * *p* < 0.05 and *** *p* < 0.001 vs. DSS group.

**Figure 7:**
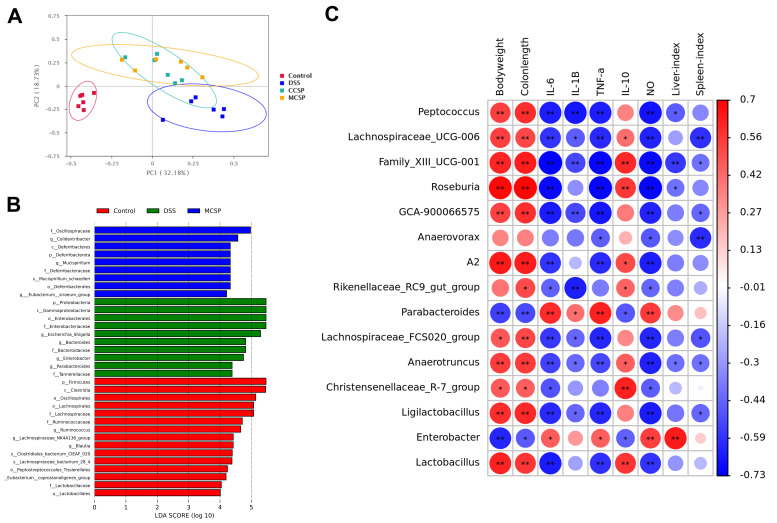
Effects of M-CSP on gut microbiota composition in UC mice. (**A**) Beta diversity and principal component analysis. (**B**) LEfSe discriminant analysis. (**C**) Microbiota–indicator correlation heatmap across groups. Data represent mean ± standard deviation (*n* = 6). * *p* < 0.05 and ** *p* < 0.01 vs. DSS group.

UC development is closely related to imbalance in the intestinal microbiota. Table S7 lists read counts for each group. As shown in Figure 6A–D, M-CSP intervention enhanced microbial diversity and richness in DSS-induced colitis mice. Moreover, PCA revealed a distinguishable difference in the gut microbiota between the Con and DSS groups (Figure 7A). At the genus level, DSS downregulated the relative abundance of *Blautia*, *Lachnospiraceae_NK4A136_group*, and *Akkermansia*, and upregulated the levels of *Escherichia-Shigella*, *Enterobacter*, and *Bacteroides* compared with the Con group. Simultaneously, M-CSP treatment reversed this relative abundance (Figure 6E). At the species level, M-CSP exhibited regulatory effects by inhibiting *Parabacteroides merdae* and promoting *Clostridiales bacterium CIEAF 020* and *Clostridium sp Culture-41* (Figure 6F). In addition, the ratio of F/B (Firmicutes/Bacteroidetes) increased from 0.12 to 2.04 after oral M-CSP administration, favoring reduced inflammation and obesity.

A linear discrimination analysis (LDA) effect scale (LEfSe) was employed to comprehensively examine the variations in gut microbiota among the groups (Figure 7B). The abundance of *Proteobacteria*, *Enterobacteriaceae*, *Enterobacteriales*, *Escherichia-shigella*, and *Bacteroides* was increased in the DSS group, whereas M-CSP treatment significantly prevented DSS-induced changes in gut microbiota. In addition, M-CSP treatment increased the abundance of *Oscillospiraceae*, *Colidextribacter* and *Deferribacteres* in DSS mice. Spearman’s correlation analysis was used to investigate the relationship between UC and alterations in microbiota composition. As depicted in Figure 7C, *Parabacteroides* and *Enterobacter* abundance was negatively correlated with IL-10 and positively correlated with IL-6, IL-1β, and TNF-α. However, *Peptococcus*, *Roseburia*, *Lachnospiraceae ucg 006,* and *Lactobacillus* showed negative associations with IL-6, IL-1β, and TNF-α, indicating their intensive association with reduced inflammation.

## 4. Discussion

During germination, seeds utilize starch and other dry matter to fuel growth and activate metabolic pathways that synthesize and integrate complex carbohydrates, such as cellulose and hemicellulose, into new cell walls, thereby increasing dietary fiber content [36]. SMF treatment promotes the accumulation of active ingredients. SMF treatment at 30 mT for 2 h significantly increased the dietary fiber content of corn sprouts. This was attributed to the enhanced activity of SDF-synthesizing enzymes during germination under SMF treatment, which promoted the conversion of IDF and hemicellulose into soluble components. Simultaneously, SMF stimulates enzymatic activities related to anabolic metabolism, accelerating the synthesis of dietary fiber in corn sprouts and elevating its total content [37]. In addition, SMF treatment promoted the synthesis and metabolism of polyphenols in corn sprouts. Germination stimulates endogenous enzymes (e.g., phenylalanine ammonia-lyase, glycosidases) that synthesize and hydrolyze bound phenolics into free forms [38]. SMF treatment further enhances enzymatic activity and facilitates the release of bound phenolics from the cell wall matrix, significantly increasing free phenolic acids. SMF treatment first activates phenylalanine ammonia lyase (PAL), the primary rate-limiting enzyme in the phenylpropanoid metabolic pathway, and enhances its activity, thereby generating sufficient cinnamic acid to provide the foundation for subsequent polyphenol synthesis and metabolism [39]. SMF treatment also enhances the activity of cinnamate 4-hydroxylase (C4H) and promotes the synthesis of coumaric acid. This process provides sufficient precursors for subsequent production of phenolic acids such as caffeic acid and ferulic acid, thereby promoting the formation of flavonoids via the phenylpropanoid metabolic pathway [40].

Using a mouse model of colitis to validate its efficacy, we observed that M-CSP played an important role in intestinal health. SMF treatment improved the nutritional quality of corn sprouts by elevating dietary fiber and polyphenol content, which were strongly associated with the health improvements observed in mice. Related studies have shown that foods rich in dietary fibers and polyphenolic compounds may reduce the incidence of colitis [41,42]. As prebiotics, dietary fiber metabolites can selectively improve the composition of intestinal microbiota, thereby increasing the levels of intestinal short-chain fatty acids, such as butyric acid, propionic acid, and acetic acid, while reducing the expression of inflammatory factors to alleviate the body’s inflammatory response [43,44]. Additionally, dietary fibers obtained from SMF treatment have been shown to exhibit stronger antioxidant activity [12]. This effect may arise because SMF treatment elevates endogenous enzymatic activity in corn sprouts, facilitating the enhanced degradation of lignin within dietary fibers, generating aromatic secondary metabolites, and boosting antioxidant capacity [45]. In addition, the antioxidant capacity of dietary fiber is related to its polyphenol content [46]. Increased content of bound polyphenols contributes to enhanced antioxidant defenses [47]. Studies have demonstrated that phenolic compounds liberated during colonic fermentation exhibit enhanced antioxidant activity compared to the oral, gastric, or small intestinal phases, resulting in corresponding elevations in plasma antioxidant capacity [48,49]. Increased polyphenol content in corn sprouts treated with SMF can effectively reduce the damage caused by oxidative stress in intestinal tissues [50,51]. Meanwhile, through the regulation of inflammation-related signaling pathways, such as NF-κb, M-CSP regulate the expression of inflammatory factors, inhibit their release, and exert anti-inflammatory effects [52,53]. The results of this study further demonstrate that SMF treatment enhances both the anti-inflammatory and antioxidant effects of corn sprouts.

Accumulating evidence indicates that the gut microbiota is integral to UC pathogenesis [54]. Multiple studies have demonstrated a significant imbalance in gut microbiota in UC [55]. In this study, M-CSP, rich in dietary fiber and polyphenols, alleviated the symptoms of DSS-induced colitis. Further analysis of intestinal microbiota revealed that M-CSP modulated the composition of gut microbiota in DSS mice. The ratio of *Firmicutes*/*Bacteroidetes* (F/B) is an important index of gut microbiota structure. In this study, DSS treatment significantly reduced F/B values, which were reversed by promoting *Firmicutes* and reducing *Bacteroidetes* after the administration of M-CSP, comprising a high content of polyphenols and dietary fiber. Specifically, *Escherichia-Shigella* and *Enterobacter* were significantly reduced after M-CSP treatment, and most of the *Aspergillus phyla* are considered opportunistic bacteria for UC [56], with *Escherichia-Shigella* being a typical genus of *Aspergillus phyla*. Previous studies have shown that the development of colitis leads to an increase in *Escherichia-Shigella* and *Bacteroides*, which disrupts intestinal barrier integrity and causes intestinal microbial dysbiosis [57,58]. Previous studies proved that *Blautia* [59] and *Akkermansia* [60,61] are probiotics that prevent damage to the gut immune barrier. Studies have indicated that anthocyanins promote the proliferation of *Akkermansia*, thereby enhancing intestinal mucosal integrity [62]. Meanwhile, the *Lachnospiraceae NK4A136 group* is among the gut bacteria that produce butyric acid, which is vital in maintaining immune homeostasis. Therefore, the findings suggest that M-CSP may prevent the development of gut microbiota dysbiosis in DSS-induced colitis mice. Overall, these results demonstrate that SMF treatment improves the content of active components of corn sprouts, and such enhancements contribute to alleviating colitis by modulating gut microbiota, reducing oxidative stress, and suppressing inflammation.

## 5. Conclusions

In this study, compared with C-CSP, M-CSP exhibited a higher content of dietary fiber and polyphenols which contributed to more effective protection against UC. The beneficial effects included preventing weight loss, maintaining intestinal tissue structure, protecting the intestinal barrier from damage, and modulating inflammatory cytokines. M-CSP maintained intestinal homeostasis and enhanced intestinal function by regulating intestinal microbiota. This study examined the effects of M-CSP on colitis and established the foundation for the development of functional foods for UC treatment.

## Figures and Tables

**Table 1:** Content of active components in different groups of corn sprout powder.

Nutrient	Corn (g/100 g)	C-CSP (g/100 g)	M-CSP (g/100 g)
Moisture	13.72 ± 0.18 ^a^	6.69 ± 0.07 ^Ab^	6.71 ± 0.32 ^Ab^
Ash	1.49 ± 0.52 ^b^	4.55 ± 0.01 ^Aa^	4.64 ± 0.21 ^Aa^
Protein	9.18 ± 0.52 ^b^	25.30 ± 0.52 ^Aa^	24.91 ± 0.11 ^Aa^
Fat	5.26 ± 0.46 ^b^	6.32 ± 0.09 ^Aa^	5.82 ± 0.11 ^Bb^
Soluble sugars	2.39 ± 0.26 ^c^	3.04 ± 0.07 ^Bb^	4.50 ± 0.28 ^Aa^
Starch	73.97 ± 1.31 ^a^	5.92 ± 0.76 ^Ab^	4.85 ± 0.69 ^Ab^
Total Dietary Fiber (TDF)	2.14 ± 0.21 ^c^	46.31 ± 0.31 ^Bb^	47.39 ± 0.09 ^Aa^
Soluble Dietary Fiber (SDF)	0.36 ± 0.05 ^c^	4.09 ± 0.03 ^Bb^	4.39 ± 0.12 ^Aa^
Insoluble Dietary Fiber (IDF)	1.78 ± 0.26 ^c^	42.21 ± 0.28 ^Bb^	43.01 ± 0.20 ^Aa^
Total phenol	0.20 ± 0.01 ^c^	1.76 ± 0.03 ^Bb^	2.33 ± 0.08 ^Aa^
Free phenol	0.03 ± 0.01 ^c^	1.50 ± 0.05 ^Bb^	1.86 ± 0.07 ^Aa^
Bound phenolics	0.17 ± 0.01 ^c^	0.26 ± 0.03 ^Bb^	0.48 ± 0.02 ^Aa^

Different lowercase letters in the same row represent significant differences between groups, and different capital letters indicate significant differences between C-CSP and M-CSP (*p* < 0.05). Data represent mean ± standard deviation (*n* = 6). C-CSP: SMF-Untreated Corn Sprout Powder; M-CSP: SMF-Treated Corn Sprout Powder.

**Table 2:** Organ index of mice in each group.

Organ	Con	DSS	C-CSP	M-CSP
Heart	4.78 ± 0.45 ^a^	5.00 ± 0.69 ^a^	5.13 ± 0.72 ^Aa^	4.53 ± 0.23 ^Aa^
Liver	44.60 ± 1.81 ^a^	40.78 ± 1.62 ^b^	41.05 ± 0.68 ^Ab^	42.46 ± 1.11 ^Ab^
Spleen	2.30 ± 0.14 ^c^	4.20 ± 0.53 ^a^	3.38 ± 0.79 ^Ab^	2.57 ± 0.24 ^Bc^
Lung	6.62 ± 0.40 ^b^	7.96 ± 1.33 ^a^	6.89 ± 0.72 ^Ab^	6.69 ± 0.19 ^Ab^
Kidney	7.81 ± 3.04 ^c^	15.30 ± 0.79 ^a^	13.78 ± 0.97 ^Aab^	11.43 ± 0.78 ^Bb^

Different lowercase letters in the same row represent significant differences between groups, and different capital letters indicate significant differences between C-CSP and M-CSP (*p* < 0.05). Data represent mean ± standard deviation (*n* = 6). Con: Control group; DSS: Dextran sulfate sodium-induced colitis model group; C-CSP: SMF-Untreated Corn Sprout Powder; M-CSP; SMF-Treated Corn Sprout Powder.

## Data Availability

The original contributions presented in the study are included in the article/Supplementary Materials. Further inquiries can be directed to the corresponding authors.

## Supplementary Materials

The following supporting information can be downloaded at: https://www.mdpi.com/article/10.3390/foods14183248/s1. Table S1. Feed formula; Table S2. Disease activity index of mice; Table S3. Histopathological score; Table S4. Primer sequence for qRT-PCR; Table S5. Effect of M-CSP on redox level in colitis mice; Table S6. Effect of M-CSP on the cytokine expression in colitis mice; Table S7. Read counts per group.

## Abbreviations

The following abbreviations are used in this manuscript:

SMF	Static Magnetic Field
UC	Ulcerative Colitis
DSS	Dextran Sulphate Sodium
CSP	Corn Sprout Powder
C-CSP	Static Magnetic Field-Untreated Corn Sprouts
M-CSP	Static Magnetic Field-Treated Corn Sprouts
H&E	Hematoxylin–Eosin staining
DAI	Disease Activity Index
IL-6	Interleukin-6
IL-1β	Interleukin-1β
TNF-α	Tumor necrosis factor-α
L-10	Interleukin-10
NO	Nitric oxide
MDA	Malondialdehyde
SOD	Superoxide Dismutase
MPO	Myeloperoxidase
COX-2	Cyclooxygenase-2
iNOS	Inducible nitric oxide synthase
NF-κb	Nuclear factor-kappa b
IκB	IkappaB
ZO-1	Zonula occludens-1
MUC2	Mucoprotein2
