# Supplementary material for: Comparative Protective Effects of Static Magnetic Field-Treated and Untreated Corn Sprouts on DSS-Induced Ulcerative Colitis in Mice: Inflammation Modulation and Gut Microbiota Regulation
Source: Foods. 2025 Sep 18;14(18):3248. doi: 10.3390/foods14183248 (PMC12470086; doi:10.3390/foods14183248)
Supplement: Supplementary file 1 [file foods-14-03248-s001.zip › foods-3812122-supplementary.pdf]

Supplementary data

Comparative Protective Effects of Static Magnetic Field-Treated and Untreated Corn Sprouts on DSS-Induced Ulcerative Colitis in Mice: Inflammation Modulation and Gut Microbiota Regulation

Jiaqi Zhao 1,2, Ye Gu 1,2, Shijie Sun 1,2, Aoran Guo 1,2, Mingzhu Zheng 1,2, Dan Cai 1,2, Ke Lin 1,* and Huimin Liu 1,2,*

1 College of Food Science and Engineering, Jilin Agricultural University, Changchun, Jilin, 130118, China

2 National Engineering Research Center of Wheat and Corn Deep Processing, Changchun 130118, China

* Correspondence: liuhuimin@jlau.edu.cn, linke@jlau.edu.cn

1. **Methods**

1.1. SMF Calibration and Treatment

Prior to each use, the magnetic field intensity was calibrated using a gaussmeter. During experiments, samples must be positioned at the central location within the instrument chamber to ensure field homogeneity and experimental reproducibility.

1.2. Extraction of free phenolics and bound phenolics in corn sprouts

Extraction of free phenolics: Take 1g of corn sprout powder, add 50% methanol at a solid-to-liquid ratio of 1:40 (g:mL), perform ultrasonic extraction for 20 min, centrifuge at 5000 rpm for 15 min, and collect the supernatant. Repeat the operation twice, combine the three supernatants, evaporate to dryness at 45°C using rotary evaporation, and reconstitute the residue with 50% methanol to a final volume of 10mL. Store protected from light at -20°C.

Extraction of bound phenolics: take the above residue, add n-hexane at a solid-to-liquid ratio of 1:40 (g:mL), shake vigorously for 5 min, and centrifuge at 4000 rpm for 15 min. Discard the supernatant and repeat the process three times. To the resulting precipitate, add 4 mL of 10% H_2SO_4, and hydrolyze in a water bath at 75°C for 1 h. After hydrolysis, add 40 mL of ethyl acetate, shake vigorously, and extract five times. Centrifuge at 5000 rpm for 15 min, combine the extraction solutions, evaporate to dryness at 45°C using rotary evaporation, and reconstitute the residue with 50% methanol to a final volume of 10 mL. Store protected from light at -20°C.

Table S1 Feed formula

Component	Normal Diet	C-CSP Diet (5%)	M-CSP Diet (5%)
Casein	200.00	187.35	187.54
L-Cystine	3.00	3.00	3.00
Corn Starch	397.50	386.30	386.62
Maltodextrin	132.00	132.00	132.00
Sucrose	100.00	100.00	100.00
Cellulose	50.00	26.85	26.30
Soybean Oil	70.00	66.99	67.04
Tert-Butylhydroquinone	0.01	0.01	0.01
Mineral Mixture	35.00	35.00	35.00
Vitamin Mixture	10.00	10.00	10.00
Choline Tartrate	2.50	2.50	2.50
C-CSP		50.00	
M-CSP			50.00

Table S2 Disease activity index of mice

Score	Weight loss（%）	Fecal character	Degree of blood in stool
0	0	Normal	Normal
1	1-5	Thin but formed stools	Occult blood
2	5-10	Meager stool	Bleeding (slight)
3	10-15	Diarrhea (mild)	Hemorrhage
4	>15	Diarrhea (water sample)	Heavy bleeding

Table S3 Histopathological score

Evaluation parameters	Characterizations	Score
inflammatory	Normal, no inflammation	0
	Mild inflammation	1
	Moderate inflammation	2
	Severe inflammation with marked inflammatory cell infiltration and extensive gland separation	3
mucosal damage	Normal	0
	Damage limited to the mucosal layer	1
	Injuries penetrate deep into the submucosa	2

	Injuries penetrate deep into the muscular and plasma layers	3
Degree of glandular damage	Normal	0
	Minimal: small amount of glandular dilatation	1
	Mild: multifocal glandular dilatation	2
	Moderate: multifocal glandular dilatation with concomitant glandular disappearance	3
Scope of impact	None	0
	A smidgen：<10%	1
	Mildly：10-25%	2
	Moderate：26-50%	3
	Conspicuous：>50%	4

Table S4 Primer sequence for qRT-PCR

Gene	Forward primer	Reverse primer
ZO-1	CTTCTCTTGCTGGCCCTAAAC	TGGCTTCACTTGAGGTTTCTG
Occludin	CACACTTGCTTGGGACAGAG	TAGCCATAGCCTCCATAGCC
Claudin-1	GATGTGGATGGCTGTCATTG	CCTGGCCAAATTCATACCTG
MUC2	ATGCCCACCTCCTCAAAGAC	GTAGTTTCCGTTGGAACAGTGAA
NF-κB p65	GCTTTGCAAACCTGGGAATA	TCCGCCTTCTGCTTGTAGAT
IκB	CCAGAGAACGAAGAGGAGCC	TCCGGTTTATTGAGGTCGGC
MPO	TGAATCCTCGATGGAATGGG	ACGGAAAGCGTTGGTGAAGA
COX-2	GAAGTCTTTGGTCTGGTGCCTG	GTCTGCTGGTTTGGAATAGTTG
iNOS	CTTGGAGCGAGTTGTGGATTGTC	TAGGTGAGGGCTTGGCTGAGT
GAPDH	AGGTTGTCTCCTGCGACT	TGCTGTAGCCGTATTCATTGTCA

Table S5 Effect of M-CSP on redox level in colitis mice

group	NO (μmol/gprot)	MDA (nmol/mL)	SOD (U/mL)
Con	9.11 ± 1.83^{b}	26.54 ± 0.98^{d}	123.10 ± 5.16^{a}
DSS	16.03 ± 1.80^{a}	56.09 ± 2.18^{a}	54.41 ± 5.73^{d}
C-CSP	12.96 ± 0.86^{b}	44.37 ± 1.01^{b}	80.84 ± 5.06^{c}
M-CSP	10.90 ± 1.30^{b}	36.89 ± 3.04^{c}	95.98 ± 6.25^{b}

Different lowercase letters in the same column represent significant differences between groups ($p<0.05$). Data are expressed as mean ± SD (n=6).

Table S6 Effect of M-CSP on the cytokine expression in colitis mice

group	IL-6 (pg/mgprot)	IL-1β (pg/mgprot)	TNF-α (pg/mgprot)	IL-10 (pg/mgprot)
Con	11.05±1.12[c]	4.44±1.21[c]	62.36±4.36[c]	31.71±2.23[a]
DSS	22.48±1.87[a]	16.20±2.01[a]	184.61±15.91[a]	13.90±1.31[c]
C-CSP	16.39±1.53[b]	8.48±2.01[b]	118.56±11.17[b]	25.03±2.22[b]
M-CSP	14.34±1.67[b]	5.15±1.27[c]	91.98±8.86[b]	27.19±2.08[b]

Different lowercase letters in the same column represent significant differences between groups (p <0.05). Data are expressed as mean ± SD (n=6).

Table S7 Read counts per group

group	RawPE
Con	108196.83±6407.33
DSS	99857±9158.08
C-CSP	102481.17±4067.80
M-CSP	110589.17±5299.31
